# Compatibility of Bt biopesticides and adjuvants for *Spodoptera frugiperda* control

**DOI:** 10.1038/s41598-021-84871-w

**Published:** 2021-03-05

**Authors:** Cicero Antônio Mariano dos Santos, Joacir do Nascimento, Kelly Cristina Gonçalves, Giovani Smaniotto, Leonardo de Freitas Zechin, Marcelo da Costa Ferreira, Ricardo Antônio Polanczyk

**Affiliations:** 1 PhD in Agricultural Entomology, Department of Plant Health, São Paulo State University (Unesp), School of Agricultural and Veterinarian Sciences, Jaboticabal, 14884-900 Brazil; 2 PhD Student in Agricultural Entomology, Department of Plant Health, São Paulo State University (Unesp), School of Agricultural and Veterinarian Sciences, Jaboticabal, 14884-900 Brazil; 3 Student in Agronomy, Department of Plant Production, São Paulo State University (Unesp), School of Agricultural and Veterinarian Sciences, Jaboticabal, 14884-900 Brazil; 4 Department of Plant Health, São Paulo State University (Unesp), School of Agricultural and Veterinarian Sciences, Jaboticabal, 14884-900 Brazil; 5 Department of Plant Health, São Paulo State University (Unesp), School of Agricultural and Veterinarian Sciences, Jaboticabal, 14884-900 Brazil

**Keywords:** Biotechnology, Microbiology, Plant sciences

## Abstract

*Spodoptera frugiperda* is a pest of economic importance for several crops with resistance reports to Bt crops and pesticides. Eco-friendly Bt biopesticides may be an alternative to chemical insecticides due to their selectivity and specificity. However, the efficacy of Bt biopesticides may be influenced by the association with other chemicals, such as adjuvants. This study evaluated the compatibility and toxicity of Bt biopesticides mixed with adjuvants for the control of *S. frugiperda*. The treatments included the association of Dipel SC and Dipel PM with adjuvants. Compatibility tests were used to evaluate the Bt mixture. Bt suspensions obtained from mixtures of Bt and adjuvants at 10^6^ and 3 × 10^8^ spores/mL^−1^ were used to evaluate *S. frugiperda* mortality and distilled water was used as the control. The addition of the adjuvant LI increased growth and sporulation, indicating compatibility with Bt biopesticides. The other adjuvants were toxic to reducing Bt growth and sporulation. Only the mixture of Bt with LI and Bt alone was effective to *S. frugiperda*. The addition of adjuvants to Bt biopesticide affect the Bt sporulation, growth and mortality.

## Introduction

*Spodoptera frugiperda* (J.E. Smith) (Lepidoptera: Noctuidae) is an economically important pest owing to its polyphagous habit, voracity^1,2^, high reproductive capacity, long adult dispersal^3^, variability among populations^4^, and multiple generations per year^5^. Typically, pesticides and transgenic *Bacillus thuringiensis* (Bt) crops are employed for the control of this pest; however, the number of reports on the resistance of *S. frugiperda* to both strategies has been increasing^6–9^, with similar observations reported in other lepidopteran pests. In this context, Bt-based biopesticides can be used as an alternative to conventional control measures, as they can replace organic pesticides because of higher specificity and fewer cases of field-evolved resistance^10,11^.

*Bacillus thuringiensis* is a gram-positive bacterium, which produces toxins during the vegetative and sporulation phases. Specifically, the proteins and Cry toxins produced during the vegetative phase are toxic to insects, mites, nematodes, and protozoans^12–16^. The mode of action of Bt involves the ingestion of the spore–crystal complex by the susceptible insect, solubilization and activation by intestinal proteases, recognition and binding to receptors in the midgut, and pore formation and cell lysis^17,18^. The multiplication of spores in the insect midgut leads to septicemia, which occurs when the intestinal pH is reduced, ultimately killing the insect^19^.

In general, Bt biopesticides are sprayed alone or in combination with adjuvants^20,21^. Adjuvant addition to spray liquids has become increasingly relevant due to the protection of droplets and active ingredients, reduction of drift and evaporation, and extension of the spread of droplets on the leaf surface^22,23^.

Nonetheless, Bt biopesticides and adjuvants must be evaluated for compatibility before use in a tank mix, given the effects of adjuvants on the physicochemical^21^ and biological characteristics of the spray liquids, such as inhibition or stimulation of the vegetative growth^24,25^, reproduction, mutation, and even inactivation of Bt spores and crystals^26^. However, studies on the effects of adjuvants on Bt biopesticides, particularly the viability and field efficiency of Bt, are lacking^27^. To this end, the objective of the present study was to evaluate the effects of adjuvants on the efficacy of Bt biopesticides against *S. frugiperda*.

## Results

### Effects on vegetative growth

**Figure 1:**
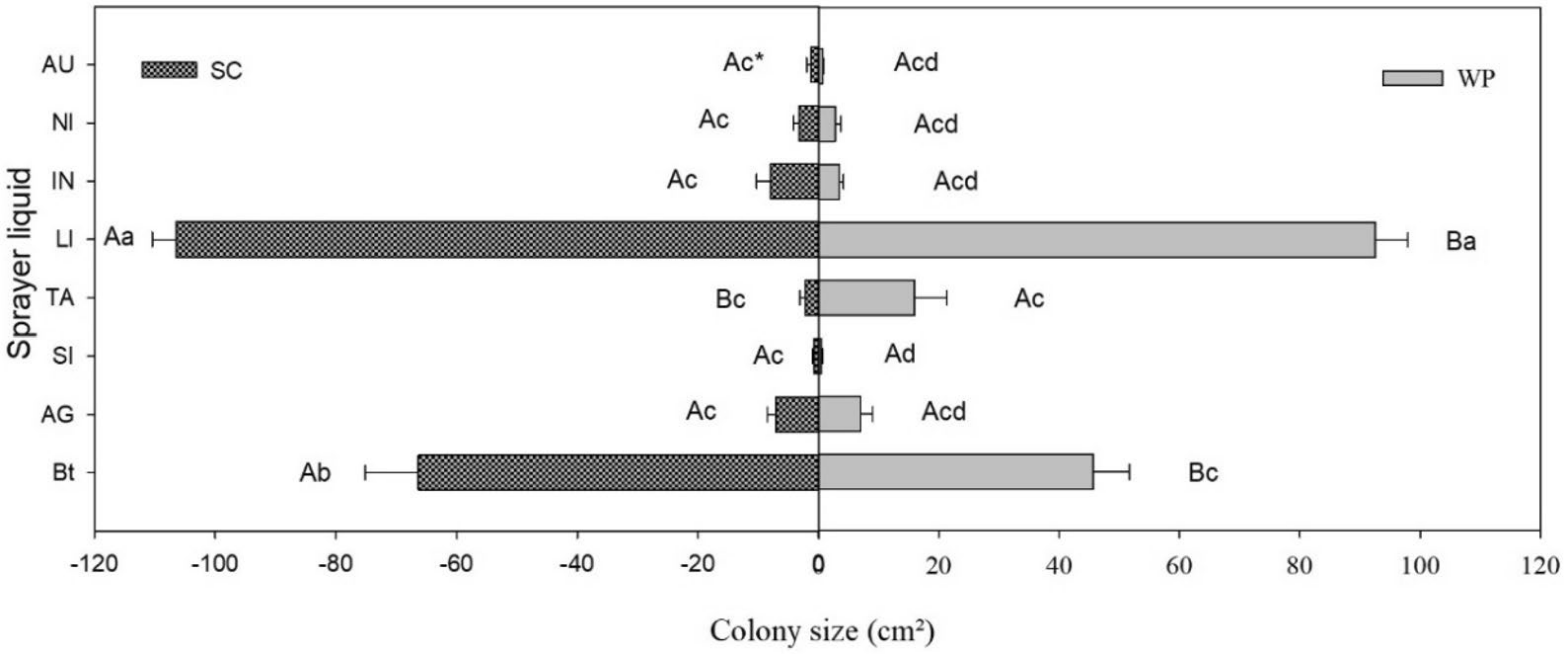
Mean size of colonies in Bt biopesticide and adjuvant mixtures. *Capital letters compare the effect of formulations. Lower case letters compare the effect of adding an adjuvant to sprays. *SC* suspension concentrate, *WP* wettable powder, *AU* AUREO, *NI* NIMBUS, *IN* IN-TEC, *LI* LI-700, *TA* TA35, *SI* SILWET, *AG* AGRAL, *Bt* Bt bioinsecticide.

The formulations containing suspension concentrate (SC) promoted colony growth compared with those containing wettable powder (WP) in almost all evaluated mixtures; however, in the mixture containing the adjuvant TA35 (TA), composed of sodium lauryl ether sulfate, the WP formulation promoted colony growth compared with the SC formulation. The mixture containing the adjuvant Li-700 (LI), composed of lecithin and propionic acid, and Bt biopesticide induced additive effect, leading to higher vegetative growth with both formulations (Fig. 1).

### Effect on sporulation

**Figure 2:**
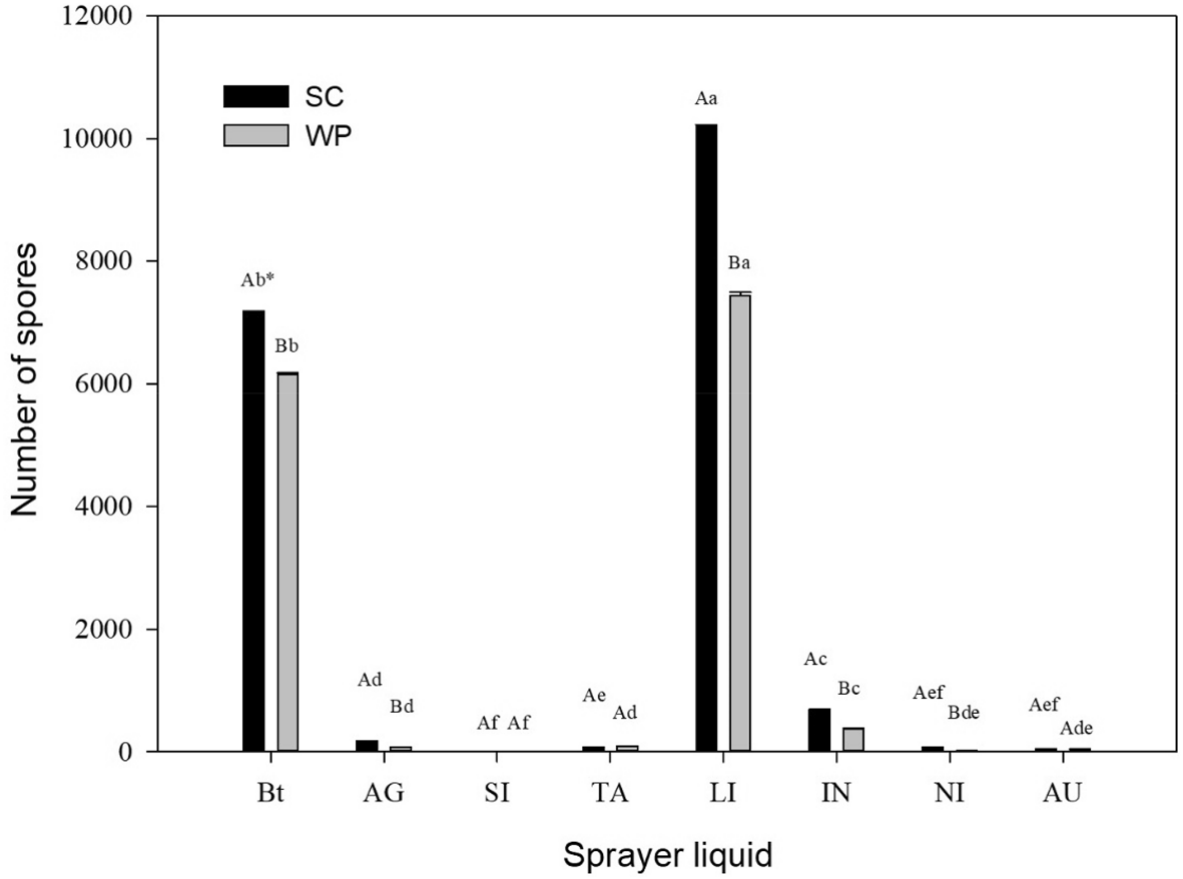
Mean size of spores in Bt biopesticide and adjuvant mixtures. *Capital letters compare the effect of formulations. Lower case letters compare the effect of adding an adjuvant to sprays. *SC* suspension concentrate, *WP* wettable powder, *AU* AUREO, *NI* NIMBUS, *IN* IN-TEC, *LI* LI-700, *TA* TA35, *SI* SILWET, *AG* AGRAL, *Bt* Bt biopesticide.

The addition of siliconized adjuvants, oils, and surfactants reduced the number of spores, whereas the addition of LI increased this number. Mixtures containing Bt bioinsecticides with adjuvants induced additive effects, leading to higher sporulation than Bt biopesticides without adjuvants (Fig. 2).

### Compatibility of Bt biopesticides and adjuvants

Regarding compatibility, almost all adjuvants added to Bt biopesticides were classified as extremely toxic. However, LI was classified as compatible (Table 1) and produced an additive effect on Bt vegetative growth and sporulation. The LI plus Bt biopesticide mixture produced more colonies and spores than Bt biopesticide alone (T value = 100).

**Table 1:** T values for classifying the effects of adjuvants on *Bacillus thuringiensis.*

Spray solution	T value	Product classification
SC + IN	3.19	Extremely toxic
SC + AG	2.18	Extremely toxic
SC + LI	146.07	Compatible
SC + TA	0.99	Extremely toxic
SC + NI	0.99	Extremely toxic
SC + SI	0.23	Extremely toxic
SC + AU	0.41	Extremely toxic
WP + IN	2.02	Extremely toxic
WP + AG	3.09	Extremely toxic
WP + LI	137.13	Compatible
WP + TA	6.99	Extremely toxic
WP + NI	1.23	Extremely toxic
WP + SI	0.22	Extremely toxic
WP + AU	0.34	Extremely toxic

*SC* suspension concentrate, *WP* wettable powder, *AU* AUREO, *NI* NIMBUS, *IN* IN-TEC, *LI* LI-700, *TA* TA35, *SI* SILWET, *AG* AGRAL, *Bt* Bt biopesticide.

### Efficiency of Bt treatment against *S. frugiperda*

**Figure 3:**
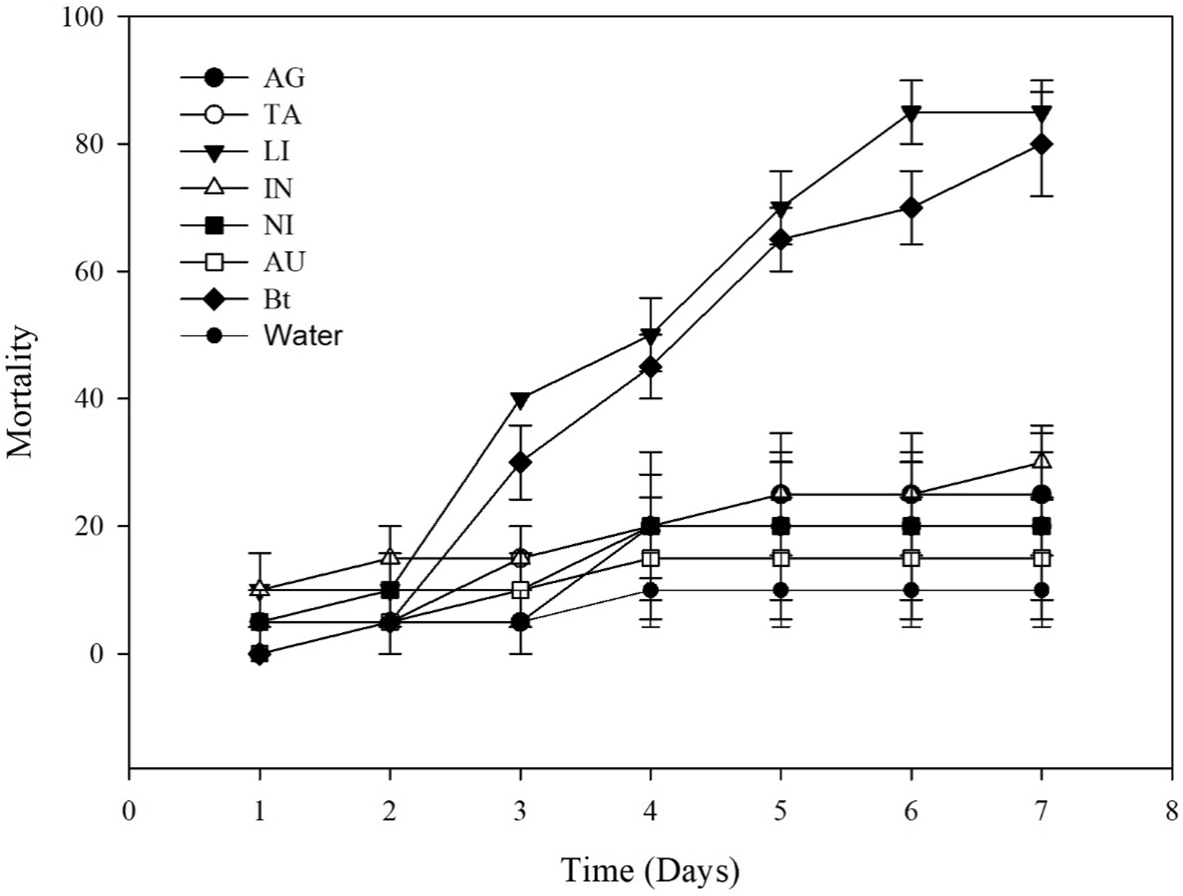
Cumulative mortality of *Spodoptera frugiperda* caused by adjuvant mixtures and Bt biopesticide in suspension concentrate (SC) formulation. Concentration of 10^6^ spores per treatment.

**Figure 4:**
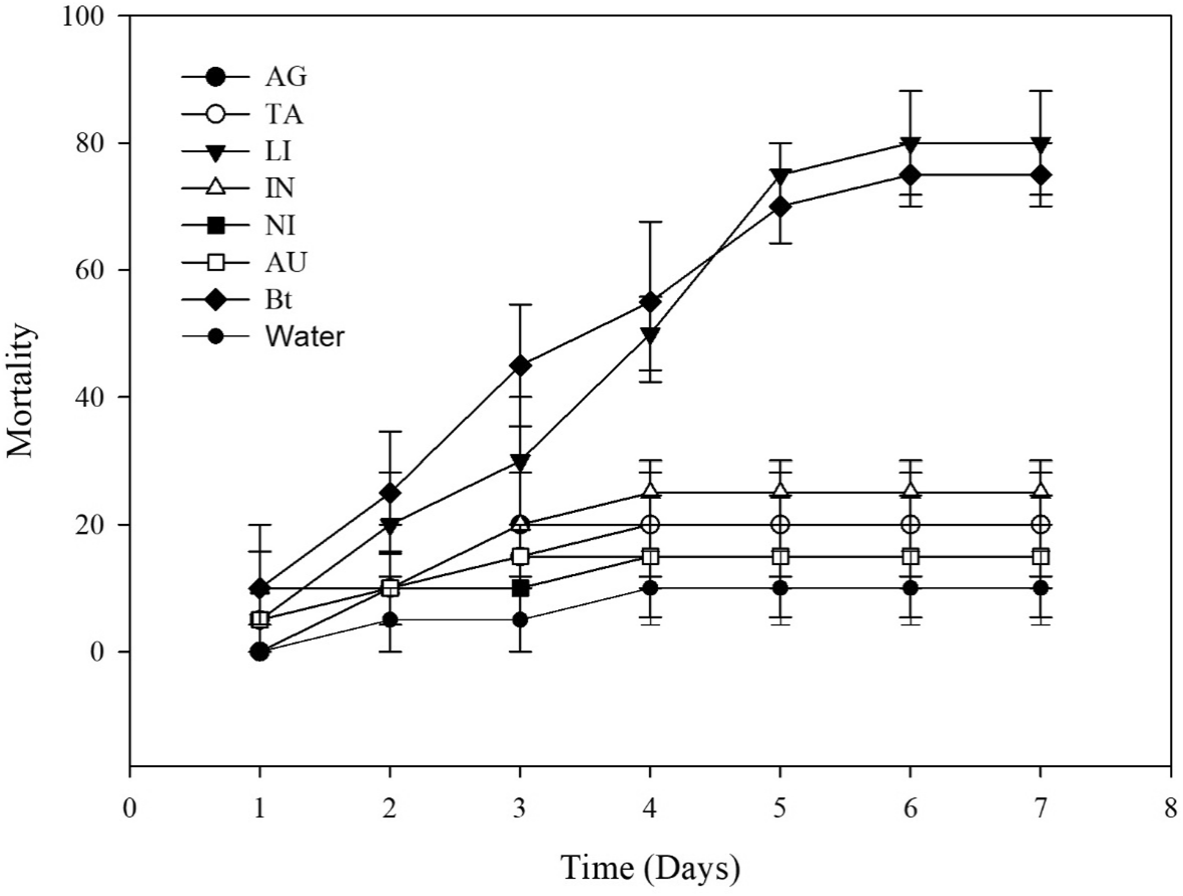
Cumulative mortality of *Spodoptera frugiperda* caused by adjuvant mixtures and Bt biopesticides in wettable powder (WP) formulation. Concentration of 10^6^ spores per treatment.

**Figure 5:**
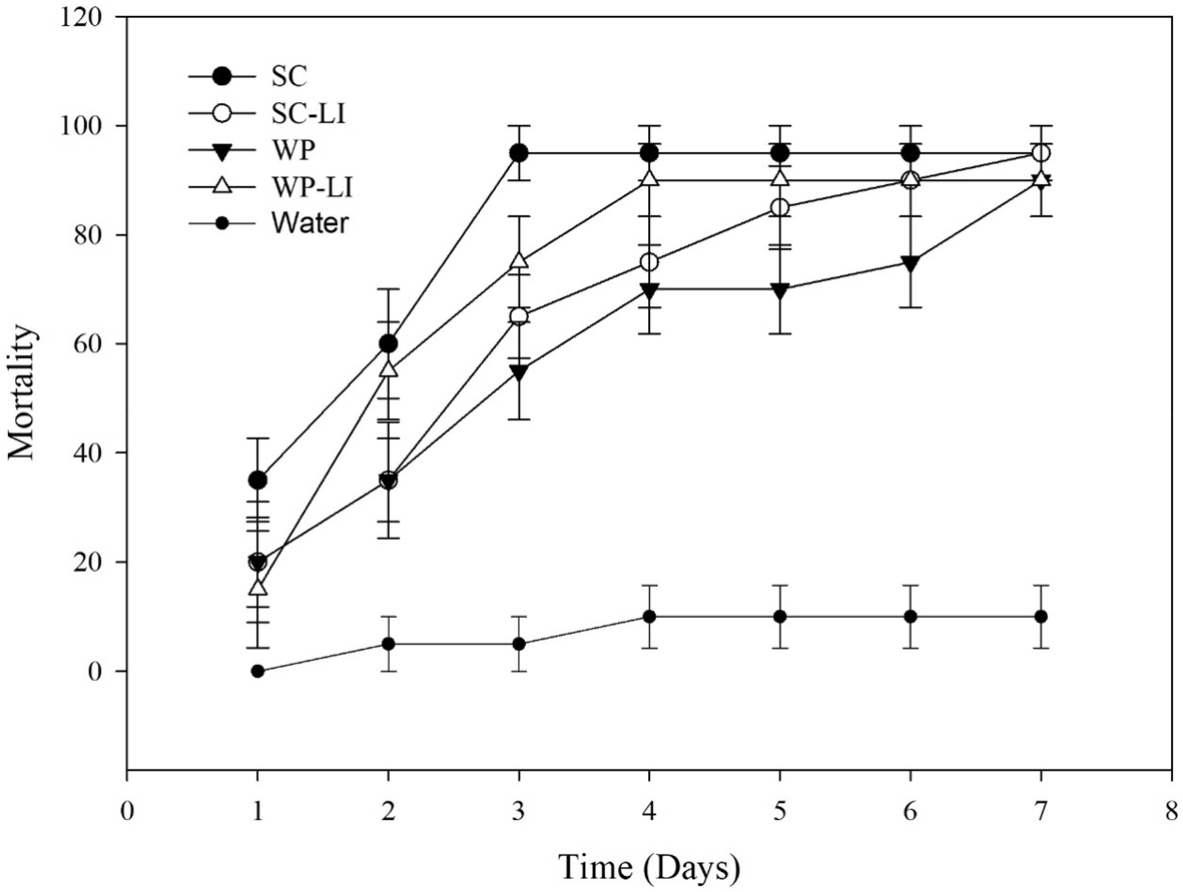
Cumulative mortality of *Spodoptera frugiperda* caused by LI mixtures and Bt biopesticides in wettable powder (WP) and suspension concentrate (SC). Concentration of 3 × 10^8^ spores per treatment.

At a concentration of 10^6^ spores·mL^−1^, both WP and SC formulations induced the highest *S. frugiperda* mortality at 5 days after treatment. The mortality rate of *S. frugiperda* following treatment with the Bt biopesticide alone or in combination with LI was over 65%; the rate following treatment with other mixtures ranged from 15 to 30% (Table 2; Figs. 3, 4). At a concentration of 3 × 10^8^ spores·mL^−1^, the mortality rate of *S. frugiperda* following treatment with Bt biopesticide alone or in combination with LI was over 90% (Fig. 5).

**Table 2:** Daily percent mortality of *Spodoptera frugiperda* following treatment with adjuvant mixtures and Bt biopesticides in Dipel SC and Dipel WP formulations.

Suspension	1D	2D	3D	4D	5D	6D	7D
Control	0a *± 0.00	5a ± 5.00	5Ab ± 5.00	10Ac ± 5.80	10Ab ± 5.80	10Ab ± 5.80	10Ab ± 5.80
Bt SC	0a ± 0.00	5a ± 5.00	30Aab ± 5.77	45Aab ± 5.00	65Aa ± 5.00	70Aa ± 5.00	80Aa ± 5.77
SC + IN	10a ± 5.77	15a ± 5.00	15Aab ± 5.00	20Abc ± 0.00	25Ab ± 5.00	25Ab ± 5.00	35Ab ± 9.57
SC + AG	0a ± 0. 00	5a ± 5.00	5Ab ± 5.00	20Abc ± 8.20	25Ab ± 12.9	25Ab ± 12.9	25Ab ± 12.9
SC + LI	10a ± 5.8	10a ± 5.80	40Aa ± 0.00	50Aa ± 5.77	70Aa ± 5.00	85Aa ± 5.77	85Aa ± 5.00
SC + TA	5a ± 5.00	5a ± 5.00	15Aab ± 5.00	20Aab ± 5.80	20Ab ± 5.80	20Ab ± 5.80	20Ab ± 5.80
SC + NI	5a ± 5.00	10a ± 5.77	10Ab ± 5.77	20Abc ± 11.5	20Ab ± 9.57	20Ab ± 9.57	20Ab ± 9.57
SC + AU	0a ± 0.00	5a ± 5.00	10Ab ± 10.0	15bc ± 9.57	15Ab ± 11.54	15Ab ± 11.54	15Ab ± 11.54
Bt WP	10a ± 5.77	25a ± 9.57	45Aa ± 9.57	55Aa ± 12.5	75Aa ± 8.16	75Aa ± 9.57	75Aa ± 9.57
WP + IN	10a ± 10.0	10a ± 10.0	20Aab ± 5.77	25Abc ± 5.00	25Ab ± 5.00	25Ab ± 8.16	25Ab ± 8.16
WP + AG	0a ± 0.00	10a ± 5.77	20Aab ± 8.16	20Ac ± 5.00	20Ab ± 5.00	20Ab ± 5.00	20Ab ± 5.00
WP + LI	5a ± 5.00	20a ± 8.16	30Aab ± 10.0	50Aab ± 5.77	70Aa ± 5.00	80Aa ± 5.77	80Aa ± 5.77
WP + TA	5a ± 5.00	10a ± 5.77	15Ab ± 5.00	20Ac ± 0.00	20Ab ± 5.77	20Ab ± 5.00	20Ab ± 5.00
WP + NI	5a ± 5.00	10a ± 10.0	10Ab ± 10.0	15Ac ± 9.57	15Ab ± 8.16	15Ab ± 5.00	15Ab ± 5.00
WP + AU	5a ± 5.00	10a ± 5.77	15Ab ± 5.00	15Ac ± 5.00	15Ab ± 5.00	15Ab ± 5.00	15Ab ± 5.00

*Means followed by the same letter are not different at a 5% significance level by Tukey test. Uppercase letters compare mortality values among formulations. Lowercase letters compare the mortality effects of treatments within each formulation.

*SC* suspension concentrate, *WP* wettable powder, *AU* AUREO, *NI* NIMBUS, *IN* IN-TEC, *LI* LI-700, *TA* TA35, SI-SILWET, *AG* AGRAL, *Bt* Bt biopesticide. Control: water.

## Discussion

The propionic acid and lecithin-based adjuvant LI promoted vegetative growth compared with other adjuvants, primarily in the form of the SC formulation. Propionic acid and lecithin serve as a major energy source for crystal synthesis, thus contributing to colony growth^28^. In other treatments, specifically those with the mixtures containing Bt biopesticide and alkylene oxide (SI), the growth of Bt colonies was low.

As recommended on the biopesticide package, the doses of LI, SI, TA, IN, and AG adjuvants were tenfold higher in the WP formulations than in the SC formulations, which explains reduced vegetative growth with the WP formulation of the LI plus Bt mixture. For adjuvants based on mineral oil (NI) and soybean oil (AU), doses recommended by the manufacturer were used. The use of lower doses of adjuvant is impracticable in the field, and higher doses of oil-based adjuvants may affect the vegetative growth and sporulation of Bt.

The inhibition or reduction of Bt vegetative growth is common when the doses of pesticides, such as thiamethoxan, malathion, and fipronil, in combination with Bt biopesticides are increased. However, at the doses recommended by the manufacturers, there are no incompatibility issues between Bt biopesticides and chemical insecticides^29,30^. Therefore, altering the recommended doses of chemicals used for preparing Bt biopesticide spray mixtures increases the probability of incompatibility.

Although pH is not considered the key determinant of Bt colony growth and sporulation, spore germination was substantially reduced in acidic media with a pH up to 5.0^21,32^, whereas spore germination was completely inhibited due to protein solubilization in alkaline media with a pH above 8^33^. In the present study, higher vegetative growth and sporulation were observed following treatment with the mixture containing Bt biopesticide and LI. The pH ranged from 3.5 for the WP formulations to 4 for the SC formulations^21^, indicating that a pH below 5 does not affect the vegetative growth of colonies and number of spores.

The addition of LI led to the production of more colonies and spores, confirming that acid media enable higher sporulation in Bt-based sprays^34^, as this bacterium produces and excretes pyruvate and acetate into the culture medium during vegetative growth. In many *Bacillus* species, these acids are derived from carbohydrate fermentation^35^ and function as intracellular carbon and energy reserves for sporulation^28^. When the minimum pH value is reached, poly-β-hydroxybutyrate is synthesized for a few hours until it reaches the maximum concentration to begin sporulation^36^. In the present study, the addition of the LI adjuvant provided an energy reserve in the form of lecithin and propionic acid for bacterial sporulation, leading to the production of more Bt spores with both formulations.

Considering their diverse effects, adjuvants may be added to Bt biopesticide mixtures, providing conditions conducive to the survival and multiplication of Bt. Additional studies on various combinations of these adjuvants are warranted to elucidate the effects of each compound and concentration as well as the use of appropriate Bt formulation.

Furthermore, all adjuvants, except LI, were classified as extremely toxic. These adjuvants suppressed the vegetative growth and sporulation of Bt, negatively affecting the efficiency of Bt biopesticides. Active ingredients and their concentration are the key variables associated with the compatibility of Bt biopesticides with adjuvants, herbicides, insecticides, fungicides, and other pesticides^25,37^. For instance, surfactants present in silicone adjuvants, adhesive surfactants, and oils used in the present study negatively affected the efficacy of Bt biopesticide against *S. frugiperda*. When added to Bt biopesticides, many pesticides inhibit Bt vegetative growth and sporulation^38–40^. This may be related to the dose of chemicals added to Bt sprays, as high doses may inhibit vegetative growth^24,29,31,41^, as well as the components present in the adjuvant formulation, ultimately affecting the efficiency of Bt biopesticides in the field.

The effects of adjuvants on Bt vegetative growth and sporulation are driven by their chemical nature and concentration, and they can act as antagonists, synergists, and additives in the biopesticide spray^42,43^. The presence of emulsifiers and other concentrated emulsifiable additives in the formulations increases their incompatibility with entomopathogens, representing an important additional factor to be controlled during the development of new commercial formulations^40^. Overall, adjuvants added to biopesticides should preserve Bt virulence and reproduction.

## Conclusions

LI presented the best compatibility with the Bt biopesticide, leading to the formation of more Bt colonies and spores under laboratory conditions. Therefore, this mixture can be used for larval control in regions where *S. frugiperda* has developed resistance to the current control measures. The other adjuvants evaluated in this study were classified as extremely toxic, adversely affecting Bt vegetative growth and sporulation.

## Methods

### *S. frugiperda* rearing

*S. frugiperda* was obtained from a mass-rearing facility at the Embrapa Sete Lagoas, Minas Gerais, Brazil. Adults were maintained in plastic cages (35 cm × 30 cm) and fed a 10% sugar solution; larvae were fed an artificial diet containing pinto bean, wheat germ, and soybean protein^44^ produced in the laboratory. Eggs were collected daily. After hatching, the larvae were individually placed in containers (diameter, 7 cm; height, 3 cm) and fed an artificial diet until pupation. The pupae were sexed and placed in cages until adult emergence. The insects were reared in an environmental chamber at 25 ± 2 °C under a relative humidity of 60%–70% and a photoperiod of 16:8 h (light:dark)^45^.

### Adjuvants used in the experiment

Cotton plants were treated to evaluate the compatibility of Bt biopesticides with adjuvants (Table 3). The cultivar ‘FIBERMAX 910’ (Bayer Crop Science) was grown in the field, and leaves were collected during the vegetative growth stage.

**Table 3:** Commercial products used to evaluate the physical and chemical characteristics of mixtures in WP and SC formulations of Dipel in terms of droplet size spectrum, contact angle, surface tension of droplets, and hydrogen and electrical conductivity.

Mixture^a^	Dose	ai of products
Dipel WP	700 g·ha^−1^	*Bacillus thuringiensis* var. *kurstaki*
Dipel WP + IN	700 g·ha^−1^ + 0.2% v/v	Bt + Nonylphenol ethoxylate (NPE)
Dipel WP + AG	700 g·ha^−1^ + 0.2% v/v	Bt + Nonylphenoxy polyethanol (NPPE)
Dipel WP + LI	700 g·ha^−1^ + 0.2% v/v	Bt + Lecithin and propionic acid (LPA)
Dipel WP + TA	700 g·ha^−1^ + 0.2% v/v	Bt + Sodium lauryl ether sulfate (SLES)
Dipel WP + NI	700 g·ha^−1^ + 750 mL·ha^−1^	Bt + Mineral oil (MO)
Dipel WP + SI	700 g ha^−1^ + 0.2% v/v	Bt + Alkylene oxide (AO)
Dipel WP + AU	700 g ha^−1^ + 375 mL·ha^−1^	Bt + Soybean oil methyl ester (SOME)
Dipel SC	625 mL·ha^−1^	Bacillus *thuringiensis* var. *kurstaki*
Dipel SC + IN	625 mL·ha^−1^ + 0.02% v/v	Bt + NPE
Dipel SC + AG	625 mL·ha^−1^ + 0.02% v/v	Bt + NPPE
Dipel SC + LI	625 mL·ha^−1^ + 0.02% v/v	Bt + LPA
Dipel SC + TA	625 mL·ha^−1^ + 0.02% v/v	Bt + SLES
Dipel SC + NI	625 mL·ha^−1^ + 750 mL·ha^−1^	Bt + MO
Dipel SC + SI	625 mL·ha^−1^ + 0.02% v/v	Bt + AO
Dipel SC + AU	625 mL·ha^−1^ + 375 mL ha^−1^	Bt + SOME

^a^All doses were recommended by the manufacturers for a spray volume of 150 L·ha^−1^.

*SC* suspension concentrate, *WP* wettable powder, *AU* AUREO, *NI* NIMBUS, *IN* IN-TEC, *LI* LI-700, *TA* TA35, *SI* SILWET, *AG* AGRAL, *Bt* Bt bioinsecticide.

### Compatibility tests of Dipel WP and SC formulations with adjuvants

A nutrient agar culture medium was used as a substrate for the compatibility tests of Bt biopesticides and adjuvants. The medium was prepared by dissolving 18.0 g of formulated nutrient agar (KASVI) in 1 L of distilled water. The mixture was autoclaved at 1 atm for 40 min. At 45 °C, when the medium had not solidified, the adjuvants were added at the recommended doses, and the mixture was homogenized with a magnetic stirrer. Then, pH was measured, and the solution was poured into 9.0 cm Petri dishes, with 10 replicates for each compatibility test. Each spray mixture was considered a single treatment. The control comprised nutrient agar culture medium without adjuvants.

After solidification of the medium, a 5.0 μL aliquot of the biopesticide suspension was inoculated at the center of the Petri dish and spread with a Drigalski spatula. The inoculated dishes were stored in a biological oxygen demand (BOD) germination chambers for 7 days for Bt development at 30 ± 2 °C under a relative humidity of 70% ± 10% and photoperiod of 12:12 h (light:dark).

A leaf area meter (model CI-202, ZEISS) was used to evaluate colony growth, and a Neubauer chamber (BOCCO, Germany) was used to count spores under a phase-contrast microscope (ZEISS AXIO) (400× magnification) in each treatment.

### Classification of biopesticide and adjuvant compatibility

Vegetative growth and sporulation following each treatment were standardized using the compatibility classification^26^ (Table 2) based on the mean percent rate of Bt sporulation and growth, using the following formula (T = 20 × [VG] + 80 × [SPO]/100), where T is the corrected value of vegetative growth and sporulation for product classification; VG is the percentage of vegetative growth relative to control; and SPO is the percentage of sporulation relative to control. The chemicals were classified as extremely toxic (T = 0–30), toxic (T = 31–45), moderately toxic (T = 46–60), and compatible (T > 60) to Bt.

### Efficiency of different concentrations of Bt suspension against *S. frugiperda*

For mortality bioassays, a 100 mL aliquot of the Bt suspension was used at a concentration of 10^6^ or 3 × 10^8^ spores·mL^−1^ depending on the sporulation rate^46^. The suspension was applied to cotton disks (diameter, 2 cm) using a Potter spray tower (Burkard Manufacturing Co., Rickmansworth, England) calibrated at 34.5 kPa with 2 mL solution of each spray^43^. The control group was treated with distilled water.

After spraying, the disks were placed on a 2.5% agar–water gel mixture (1 mL·cell^−1^) in plastic containers (diameter, 7 cm; height, 3 cm). Cotton leaf disks were separated from the agar–water layer using a filter paper disk^47^. At 1 h after applying the solutions, 20 s-instar larvae were distributed in 10 replicates for each treatment (16 treatments + control). Two second-instar *S. frugiperda* larvae were placed on each leaf disk using a thin brush. The control group received distilled water at a volume equivalent to the suspensions.

The material was stored in a BOD incubator at 25 ± 0.5 °C under 65 ± 10% relative humidity and 12-h photophase. All treatment groups were evaluated daily for 7 days after bacterial inoculation.

### Statistical analysis

Colony growth and spore number were analyzed in a 2 × 8 factorial scheme (Dipel formulation vs. adjuvants). Larval mortality was analyzed in a 2 × 8 × 7 + 1 factorial scheme (Dipel formulation vs. adjuvants vs. evaluation time + control). The obtained data were subjected to variance analysis, and when significant, the means were compared using Tukey test at a 5% probability level in SAS User 9.4.

## Data Availability

The datasets generated and/or analyzed during the current study are available from the corresponding author upon reasonable request.
